# Complete chloroplast genome sequence of *Syzygium samarangense* (Myrtaceae) and phylogenetic analysis

**DOI:** 10.1080/23802359.2022.2080022

**Published:** 2022-06-10

**Authors:** Xiuqing Wei, Liang Li, Ling Xu, Xijuan Zhang, Lihui Zeng, Jiahui Xu

**Affiliations:** a College of Horticulture, Fujian Agriculture and Forestry University, Fuzhou, China; b Fruit Research Institute, Fujian Academy of Agricultural Sciences, Fuzhou, China

**Keywords:** *Syzygium samarangense*, wax apple, chloroplast genome, phylogenetic relation

## Abstract

*Syzygium samarangense* (Blume) Merr. et Perry, 1938, commonly known as wax apple, is a Myrtaceae species that is known for its unique fruit shape, flavorful and colorful fruits, medicinal value and increasing economic relevance. In this study, we reported the complete chloroplast genome sequence of *S. samarangense*. The complete genome is 159,109 bp in length with a quadripartite structure containing two single copy regions, a Large Single Copy region (LSC, 88,155 bp) and a Small Single Copy region (SSC, 18,796 bp) separated by Inverted Repeat regions (IRs, 26,079 bp). The GC content was 37.0%. It encoded 126 genes, including 81 protein-coding genes, 37 transfer RNA genes, and 8 ribosomal RNA genes. The phylogenetic relationships of 20 species inferred that all *Syzygium* species formed a single cluster belonging to Syzygieae tribe. Our results offer insights into the evolutionary relationship of *S. samarangense* within Myrtaceae, indicating a closer relationship between *S. samarangense* and *S. forresti*i.

Wax apple (*Syzygium samarangense* (Blume) Merr. et Perry, 1938) is a Myrtaceae species native to Malaya to the Andaman and the Nicobar Islands (Morton 1987), and has become economically important in Asia such as Malaysia, Indonesia, Thailand, and China (Vara-ubol et al. 2006; Moneruzzaman et al. 2015). The extract of *S. samarangense* fruits has therapeutic value against diabetic progression (Shen et al. 2013; Shen and Chang 2013) and human colon cancer (Simirgiotis et al. 2008). By nuclear ribosomal DNA and chloroplast markers, the evolutionary history of some groups of Myrtaceae such as *Myrteae* (Vasconcelos et al. 2017; Bernardes et al. 2018), *Eugenia* (Mazine et al. 2014) and *Myrcia* (Staggemeier et al. 2015) has been investigated. However, there are only few phylogenetic and evolutionary studies based on chloroplast genomes in the case of *Syzygieae* (Asif et al. 2013; Zhang et al. 2019). In this study, the complete chloroplast genome of *S. samarangense* was assembled and subjected to phylogenetic analysis, to provide more information on better understanding about Myrtaceae family.

The specimen of *S. samarangense* was deposited at the Field GenBank for wax apple of Fujian Academy of Agricultural Sciences, Fujian province, China (http://www.faas.cn/cms/html/gsyjs/index.html; Coordinates: 26°7′53″N; 119°20′6″E; Jiahui Xu, xjhui577@163.com) under the voucher number GPLWFJGSS0058. Total genomic DNA was extracted from the fresh leaves following the manufacturer’s protocol of Hi-DNAsecure Plant Kit (Tiangen Biotech, Beijing, China). The qualified libraries were constructed and performed with the Illumina Hiseq 2500 platform at Genepioneer Biotechnologies Inc. (Nanjing, China), and all the raw reads were trimmed by Fastqc. The complete chloroplast genome was assembled by NOVOPlasty (Dierckxsens et al. 2017) until a circular genome formed, with published *S. cumini* species (NCBI Accession No. GQ870669.3) as references. The chloroplast genome annotation was performed using the CpGAVAS (Liu et al. 2012) and then manually corrected. The complete chloroplast genome of *S. samarangense* was submitted to NCBI (Accession No. MW698694).

The complete chloroplast genome of *S. samarangense* was 159,109 bp in length, with a typical quadripartite circular structure containing a pair of inverted repeat regions (IR; 26,079 bp), large single copy region (LSC; 88,155 bp) and small single copy region (SSC; 18,796 bp). The GC content of the *S. samarangense* chloroplast genome was 37.0%. A total of 126 genes were predicted, including 81 protein-coding genes, 37 tRNA genes, and 8 rRNA genes. There were 17 intron-containing genes identified, in which eight are distributed over LSC region, one in SSC region, and eight in IR regions. Besides, 34 long repeats sequences were detected. Among them, 18 repeats were found in the LSC (11), SSC (4), IR (3) regions, and the remaining 16 repeats were all across two regions.

To investigate the evolutionary relationship of *S. samarangense* within Myrtaceae, eighteen representative species of Myrtaceae family were selected, with two species (*Carica papaya* and *Henriettea barkeri*) as outgroups. Complete chloroplast genome sequences were initially aligned using MAFFT (Katoh and Standley 2013) and then visualized and manually adjusted using BioEdit (Hall 1999). The maximum-likelihood analyze was conducted by using RaxML-HPC2 on TG ver. 7.2.8 on the Cipres web server (Miller et al. 2010) to reconstruct the phylogenetic tree. The results showed that eleven species with dry fruits were clustered together to Eucalypteae tribe (Thornhill et al. 2015). Seven species with fleshy fruits were clustered together, in which *S. samarangense*, *S. forrestiiand* and *S. cumini* formed a single cluster belonging to Syzygieae tribe (Figure 1). This result supported the position that the emergence of fleshy fruit in the Syzygieae tribe occurred from a dry ancestral fruit form (Balbinott et al. 2022). Our results will provide valuable data for further phylogenomic study of Myrtaceae species.

**Figure 1.**
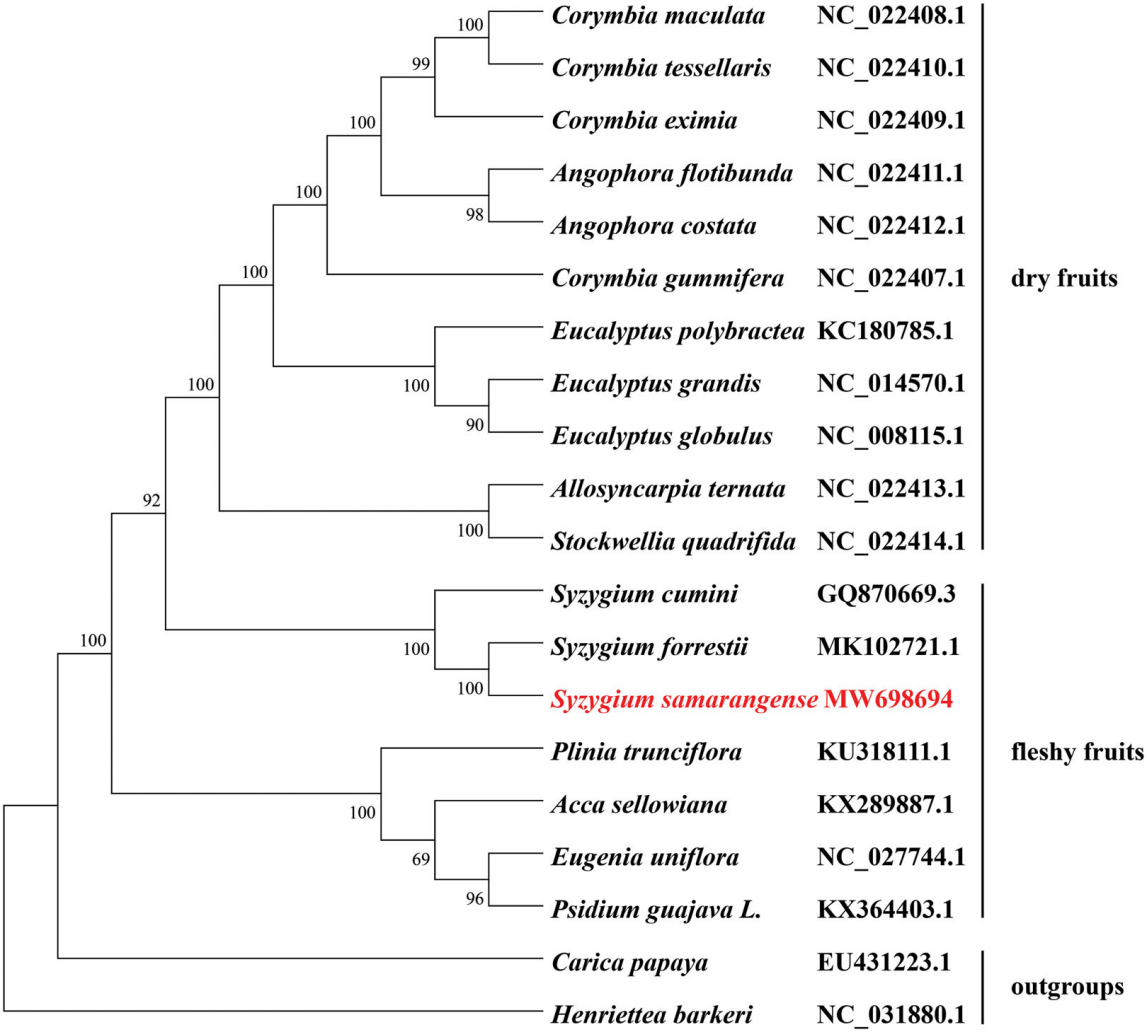
The maximum-likelihood phylogenetic tree of *S. samarangense* and other 19 related species based on the complete chloroplast genome sequences. *Carica papaya* and *Henriettea barkeri* were served as the outgroup. Numbers at nodes represent bootstrap percentage values from 1000 replicates.

## Ethical approval

In this study, the plant materials (*Syzygium samarangense* ‘Tub Ting Jiang’) were collected and studied in accordance with the Agriculture Industry Standard of China ‘Descriptors standard for tropical crops germplasm-Wax apple (NY/T 3810-2020)’ and relevant Chinese regulations.

## Author contributions

XW and JX obtained the funding support. LZ and JX conceived and designed the experiments. XW, LX and XZ collected and prepared plant materials. XW, LX and LL performed the experiments. XW and LL analyzed and interpreted the data. XW drafted the manuscript. LL revisited it critically. JX and LZ final approve the manuscript to be published. All authors agree to be accountable for all aspects of this work.

## Data Availability

The genome sequence data that support the findings of this study are openly available in GenBank of NCBI at (https://www.ncbi.nlm.nih.gov/) under the accession MW698694. Raw Illumina data were deposited in the NCBI Sequence Read Archive (SRA: SRR17364499, BioProject: PRJNA607337, and Bio-Sample: SAMN14130528).
